# Systematically characterizing and prioritizing chemosensitivity related gene based on Gene Ontology and protein interaction network

**DOI:** 10.1186/1755-8794-5-43

**Published:** 2012-10-02

**Authors:** Xin Chen, Wei Jiang, Qianghu Wang, Teng Huang, Peng Wang, Yan Li, Xiaowen Chen, Yingli Lv, Xia Li

**Affiliations:** 1 College of Bioinformatics Science and Technology, Harbin Medical University, Harbin, 150081, China

## Abstract

**Background:**

The identification of genes that predict in vitro cellular chemosensitivity of cancer cells is of great importance. Chemosensitivity related genes (CRGs) have been widely utilized to guide clinical and cancer chemotherapy decisions. In addition, CRGs potentially share functional characteristics and network features in protein interaction networks (PPIN).

**Methods:**

In this study, we proposed a method to identify CRGs based on Gene Ontology (GO) and PPIN. Firstly, we documented 150 pairs of drug-CCRG (curated chemosensitivity related gene) from 492 published papers. Secondly, we characterized CCRGs from the perspective of GO and PPIN. Thirdly, we prioritized CRGs based on CCRGs’ GO and network characteristics. Lastly, we evaluated the performance of the proposed method.

**Results:**

We found that CCRG enriched GO terms were most often related to chemosensitivity and exhibited higher similarity scores compared to randomly selected genes. Moreover, CCRGs played key roles in maintaining the connectivity and controlling the information flow of PPINs. We then prioritized CRGs using CCRG enriched GO terms and CCRG network characteristics in order to obtain a database of predicted drug-CRGs that included 53 CRGs, 32 of which have been reported to affect susceptibility to drugs. Our proposed method identifies a greater number of drug-CCRGs, and drug-CCRGs are much more significantly enriched in predicted drug-CRGs, compared to a method based on the correlation of gene expression and drug activity. The mean area under ROC curve (AUC) for our method is 65.2%, whereas that for the traditional method is 55.2%.

**Conclusions:**

Our method not only identifies CRGs with expression patterns strongly correlated with drug activity, but also identifies CRGs in which expression is weakly correlated with drug activity. This study provides the framework for the identification of signatures that predict in vitro cellular chemosensitivity and offers a valuable database for pharmacogenomics research.

## Background

Chemotherapy serves as a general defense against a large number of malignancies. However, only a portion of patients favorably respond to chemotherapy; drug efficacy and adverse drug reactions vary widely among patients [1-3]. Thus it is important to predict chemotherapy response prior to treatment and to select alternative treatment regimens for chemotherapy-resistant patients. A number of potential biomarkers have been identified in previous studies and utilized for patient specific chemotherapy selection [4]. Gene expression profiles of patients pre-treatment have the potential capability to predict responses to chemotherapy; for example, *ERCC1* activation is critical in the generation of cisplatin resistance [5]. Asparagine synthetase protein expression measured by immunoassay is a predictor of L-asparaginase activity in ovarian cancer cell lines [6]. Ovarian cancer cell lines that express low ASNS protein levels are generally more sensitive to L-ASP treatment. The expression level of p27 is also a potential candidate predictor for patient selection for rapamycin analogs-based therapy [7]. The National Cancer Institute has used a panel of 60 diverse human cancer cell lines (NCI 60 cell line) (http://genome-www.stanford.edu/nci60/index.shtml) for drug-related research [8]. It was reported that proteomic data solved pharmacologic issues more directly than genomic data [9]. For NCI 60, protein expression levels have been measured for 52 antibodies using reverse-phase protein lysate microarrays [10]. The limited number of proteins restricts identification of chemosensitivity proteins.

Some researchers have devised methods to identify chemosensitivity related genes (CRGs) based on the correlation of gene expression data and drug activity within the NCI 60 dataset [11-14]. Mariadason et al. identified CRGs for 5-fluorouracil (5-FU) by calculating the correlation coefficient of gene expression and 5-FU activity. The 50 most highly correlated genes were used to predict the response to 5-FU [15]. Szakacs et al. coupled gene expression and drug activity with bootstrap analysis to identify gene-drug pairs in which the gene potentially predicts resistance to the drug [16]. Lorenzi et al. reported that correlation coefficient of some drug-gene was not high (*r* = −0.21). The gene would not be regarded as CRG based on correlation analysis. However, aspargine synthetase was able to predict sensitivity of L-ASP [6]. However, Researchers have developed additional computational methods based on gene expression. Staunton et al. substituted correlation with t-statistics and applied 10-fold cross-validation to define classifiers for each of 232 compounds [17]. Gao et al. identified CRGs by integrating gene expression and transcription factor binding data [18]. Bayesian networks have identified CRGs by integrating different types of data such as gene expression and ChIP-chip data [19]. Although these methods provide vital information regarding CRGs, they consider individual genes in isolation rather than in the context of their functional interactions. In fact, genes are not functionally independent; they work in synergy to perform certain biological functions, such as biological processes, molecular function, complexes or pathways [20-22]. Moreover, it has been reported that chemosensitivity does not appear to be determined by the expression of a single gene [23]. Prediction of CRGs with gene sets is indeed a much more robust method compared to single gene measurement [24]. Taken together, these findings indicate that it is warranted to comprehensively explore biologically significant CRGs by not only considering the correlation between drug activity profiles and gene expression profiles, but by investigating the functional interactions of genes; this could potentially broaden the current understanding of chemosensitivity by elucidation of the context of a functional gene set.

Analyses of protein-protein interaction networks (PPINs) have revealed that genes with high betweenness centrality may be common predictive markers of chemosensitivity [25]. Sensitivity to a variety of compounds may be also influenced by certain aspects of Gene Ontology (GO) functionality, such as cell death, NADH dehydrogenase activity, ABC transporter, cell adhesion, G-protein coupled receptor protein signalling and macromolecule metabolism [16,24,26-29]. Previous studies have identified disease genes, radioresistance genes and drug target genes based on Gene Ontology and protein-interaction networks [30-32].

In this study, we proposed a novel method to identify CRGs by integrating information of Gene Ontology, protein interaction network, drug activity profile and gene expression profile. We documented 150 drug-CCRG pairs (curated chemosensitivity related gene) from 492 published papers. Most of the GO terms enriched by CCRGs were related to chemosensitivity and these terms were more similar to each other than random GO terms. Moreover, network analysis indicated that CCRGs exhibited a higher degree and betweenness centrality than random genes. Thus, we constructed an initial drug-candidate CRG network that included two types of nodes: drug nodes, in which activity data were available, and gene nodes in which expression data were available in NCI 60 cell lines. Edges of the network were weighted by Pearson’s correlation coefficient (*PCC*) between gene expression and drug activity. We then pruned the network using CCRGs’ enriched GO categories and the CCRG network characteristics. Using this method we obtained a database of predicted drug-CRGs.

## Methods

**Figure 1:**
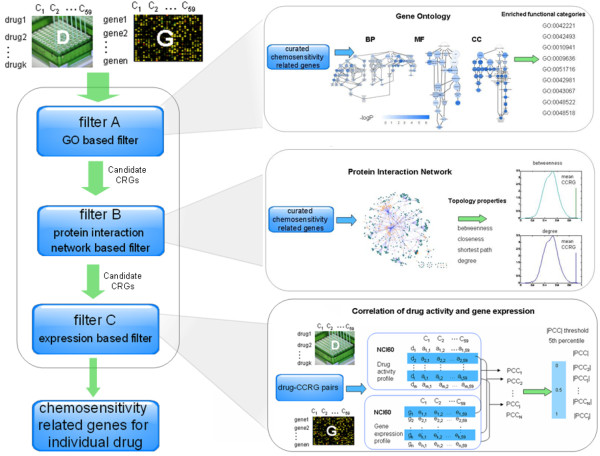
**Outline of the proposed method.** Firstly, we manually curated a compendium of curated chemosensitivity related genes (CCRGs) from published papers. Then we selected genes on the microarray that had same enriched GO categories and network characteristics with the CCRGs. These genes were considered as candidate CRGs. To get CRGs for each drug, we further filtered the initial drug-candidate CRG network based on *PCC* of drug-CCRGs. Filter A is based on Gene Ontology. We characterized CCRG using GO enrichment analysis with Fisher Exact Test. We considered three aspects of GO: biological process (BP), molecular function (MF), and cellular component (CC). p represents the enrichment significance. If enriched *p* value is smaller than 0.01, CCRGs are significantly enriched in the GO term. Moreover, we investigated that whether CCRGs exhibited functional consistency. We compared the functional similarity of CCRG enriched GO terms to randomly selected gene enriched GO terms. We found that CCRG enriched GO terms exhibited higher similarity scores compared to randomly selected genes. Thus, we regarded all genes in the enriched GO terms as candidate CRGs. Filter B is based on protein interaction networks. We analyzed several network features such as degree and betweenness centrality in six PPINs. Degree and betweenness centrality were selected as network features to prioritize CRGs. The green curve represents betweenness centrality of random genes, and the vertical green line is the betweenness centrality of CCRGs. The blue curve represents degree of random genes, and the vertical blue line is the degree of CCRGs. Filter C is based on gene expression. The majority of drug-CCRGs exhibit a low correlation between gene expression and drug activity. We ranked the absolute *PCC* of all drug-CCRG pairs in ascending order and set the *PCC* threshold as 5th percentile of all *PCC*s.

An overview of the workflow of the proposed method is shown in Figure 1. It includes four steps: 1) extensive literature survey and manually curated compendium of drug-CCRG pairs. 2) characterization of CCRGs based on Gene Ontology (GO) categories and filtering of candidate CRGs using these categories.3) characterization of CCRG networks. CCRGs exhibited higher betweenness centrality and degree compared to random genes. Based on network features, we further filtered the candidate CRGs after step 2. In Step 4 we further refined the drug-candidate CRG pair using the Pearson’s correlation coefficient between gene expression and drug activity. After performing these four steps, we finally identified CRGs for each drug; thus, researchers will be able to conduct follow-up studies on specific drugs and genes of interest. In the manuscript, drug-CCRG specifically refers to “drug-curated chemosensitivity related gene”.

### Curating drug-CCRG pairs

We searched the PubMed database with a list of keywords, such as ‘drug/compound/chemical/small molecule’ and ‘sensitive/sensitivity/resistant/resistance/response’ in the title/abstract, and using ‘National Cancer Institute’ and ‘gene/transcript/protein’ in any field of the literature. The drug-CCRG pairs were derived from experimental studies of NCI 60 cell lines (RT-PCR, siRNA, crystallographic data, etc.); of the 492 retrieved published papers, 150 pairs of drug-CCRG were documented, including 64 drugs and 94 genes. Each entry in the database contained detailed information on a drug-CCRG relationship, including the general name of the drug, gene symbol of CCRG, the cell line where the relationship was documented, literature ID in the NCBI PubMed database, and a brief description of the drug-CCRG relationship. For example, over-expression of Macrophage inhibitory cytokine-1 (*MIC-1*) predicted sensitivity of ribotoxic anisomycin. The annotated drug-CCRG table is supplemented in Additional file 1.

### Drug activity data and gene expression data

The National Cancer Institute's NCI 60 cell line panel is the most extensively characterized set of cells. These 60 human tumor cell lines are derived from patients with leukemia, melanoma, lung, colon, central nervous system, ovarian, renal, breast and prostate cancers. The analysis is presented in terms of drug activity data and microarray-based gene expression profiles of the NCI 60 cell lines.

The drug activity data we utilized included 4463 drugs [33]. Drug activities were recorded across the 60 human cancer cell lines using the logarithm of GI50 to base 10 (lgGI50). GI50 is the concentration required to inhibit cell growth by 50% compared with untreated controls. The activity profile of an agent consists of 60 such activity values, one for each cell line.

NCI 60 cell lines have been subjected to DNA and RNA microarray analysis. We utilized gene expression RNA profile data [12] (Affy-U133A, GCRMA-normalized), downloaded from the CellMiner database [34]; it comprises expression patterns of 22283 probes in NCI 60 cell lines.

### Correlation of drug activity and gene expression

Among the original 4463 drugs, 19 drugs were discarded because their activity data were missing in more than 80% of the NCI 60 cell lines. Thus the total number of drugs we analyzed in this study was 4444. *D* represents drug activity profile of the NCI 60 cell lines, each row represents a drug and each column represents a cell line, each element *a*_*ij*_ represents the drug activity (GI50) of drug *d*_*j*_ in cell line *C*_*j*_, *i* = 1,2,…,4444, *j* = 1,2,…,59. *G* represents the gene expression profiles of the NCI 60 cell lines, each row represents a gene and each column represents a cell line, each element *e*_*ij*_ represents the expression level of gene *g*_*i*_ in cell line *C*_*j*_, *i* = 1,2,…,12633. The total number of genes we analyzed in the manuscript was 12633.

In filter C based on gene expression, we characterized drug-CCRG using Pearson’s correlation coefficient (*PCC*).

$$PCC_{X,Y}=\frac{cov\left(X,Y\right)}{\delta_{X}\delta_{Y}}=\frac{E\left(\left(X-\mu_{X}\right)\left(Y-\mu_{Y}\right)\right)}{\delta_{X}\delta_{Y}}$$

where *E* is expectation, cov is covariance, and *X*, *Y* represent a drug and a gene, respectively. *δ*_*X*_^2^ = *E*(*X*^2^) − *E*^2^(*X*), *δ*_*Y*_^2^ = *E*(*Y*^2^) − *E*^2^(*Y*).

For drug-CCRG pair d2-g1, we calculated the *PCC* between drug activity of d2 and gene expression of g1 in the NCI 60 cell line. Similarly, we calculated *PCC* of other drug-CCRG pair. We ranked the absolute *PCC* of all N drug-CCRG pairs in ascending order and set the *PCC* threshold as the 5th percentile of N *PCC*s. Thus, 95% of drug-CCRGs were detected using this threshold.

### Constructing the initial drug- candidate CRG network

The initial drug-candidate CRG network includes two types of nodes: drug nodes, all the drugs with available activity data, and gene nodes with available expression data in NCI 60 cell lines. The edges of the network are weighted by Pearson’s correlation coefficient (*PCC*) between gene expression and drug activity. For some drugs, their activity data are unavailable and represented by NaN. We calculated *PCC* in the cell lines whose activity data are non-NaN.

### GO enrichment using fisher exact test

Fisher Exact test was adopted to measure the gene enrichment in annotation terms [35]. See details in Table 1.

$$\begin{array}{l} P=\frac{\left(\begin{array}{l} a+b\\ \;a \end{array}\right)\left(\begin{array}{l} c+d\\ \;c \end{array}\right)}{\left(\begin{array}{l} \;n\\ a+c \end{array}\right)}\\ \;=\frac{\left(a+b\right)!\left(c+d\right)!\left(a+c\right)!\left(b+d\right)!}{a!b!c!d!} \end{array}$$

where *n* = (*a* + *b* + *c* + *d*), *a* was the total number of user genes annotated in a GO term; *b* was the number of genes annotated in this GO term; *c* was the number of user genes not annotated in this GO term; *d* was the number of background genes not annotated in this GO term. If *p* ≤ 0.01, we hypothesized that the user gene lists were specifically associated (enriched) in this GO term. We considered all three ontologies: biological process (BP), molecular function (MF) and cellular component (CC). We limited the enriched GO term to depth 5 of GO according to DAVID [36,37].

**Table 1:** Illustration of Fisher Exact test

	**User Genes**	**Genome**
In GO term	*a*	*b*
Not In GO term	*c*	*d*

### Protein-protein interaction network

A number of publicly available human protein-protein interaction databases have become an important resource for the investigation of biological networks. PPI (protein-protein interaction) data in Human Protein Reference Database (HPRD) [38] are experimentally derived and manually extracted from the literature by expert biologists who read, interpret and analyze the published data. We downloaded protein interaction data from HPRD on the website http://www.hprd.org/download. The number of binary non-redundant human PPIs is 36687 in HPRD. The number of genes annotated with at least one interaction is 9408. We utilized “MatlabBGL” toolbox (http://dgleich.github.com/matlab-bgl/) and R package “igraph” to calculate network scores [39].

### Characterizing CCRG properties in PPIN

The degree of a gene is the number of its neighborhood genes in PPI network. One gene with high degree, termed a hub gene, plays a key role in maintaining the interactions between this gene and its neighborhood genes.

Betweenness centrality of one gene *g* is calculated as following: $B_{i}=\sum_{s\neq i\neq t}\frac{\delta_{\mathrm{st}}\left(i\right)}{d_{\mathrm{st}}}\text{,}$

Where nodes *s* and *t* are nodes in the network different from node *i* in PPI network, *d*_*st*_ denotes the number of shortest paths from *s* to *t*, *δ*_st_(*i*) is the number of shortest path from *s* to *t* that *i* lies on. For two genes *s* and *t*, the ratio is the number of shortest path that *g* lies on relative to all the possible shortest paths between genes *s* and *t*. The sum of the ratio of all gene pairs is betweenness centrality of gene *g*. If one gene exhibits high betweenness centrality, it is likely to play a vital role in gene communication and is termed a bottleneck gene.

### *Q* statistics to integrate ranks from multiple data resources

The receiver operating characteristic (ROC) curve was used to assess the performance of the two methods: the proposed method that integrates gene expression and functional interaction, and the other method based on gene expression. We ranked all CRGs in both methods and determined whether CCRGs ranked at the top of the list. Each gene was ranked in the order of degree and betweenness centrality, respectively. Next, we utilized *Q* statistic to integrate the two ranks into a final rank. The details are described as follows: $Q\left(r_{1},r_{2},\ldots ,r_{N}\right)=N!V_{N,}V_{0}=1,V_{k}={\sum_{i=1}^{k}-1}^{i-1}\frac{V_{k-i}}{i!}r_{N-k+1}^{i}$, where *r*_*i*_ is the rank ratio for data source *i*, *N* is the number of data sources used, and *r*_*0*_ = 0. In the proposed method, *N* = 2.

## Results

### Correlation-based analysis of the drug-CCRG pairs

**Figure 2:**
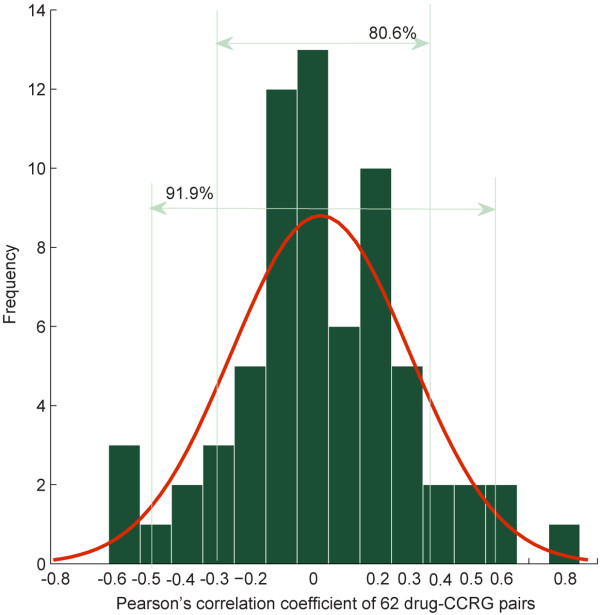
**The distribution of Pearson’s correlation coefficient *(PCC) *of drug-CCRG pairs.** The x-axis represents *PCC*, the y-axis represents the frequency of a certain *PCC*. The value 80.6% indicates that drug-CCRG pairs whose *PCC* range from −0.3 to 0.3 accounts for 80.6% of all drug-CCRG pairs; 91.9% indicates that drug-CCRG pairs whose *PCC* range from −0.5 to 0.5 accounts for 91.9% of all drug-CCRG pairs.

Previous studies identifying CRGs have been generally based on correlation of gene expression and drug activity. A gene with expression highly correlated to drug activity is regarded as a candidate CRG for the drug. Thus, we initially investigated whether CCRGs were highly correlated with their interactive drugs. Of the 150 pairs of drug-CCRG, 62 pairs were available for correlation analysis. We evaluated the *PCC* between drug activity and gene expression for drug with drug activity and genes with expression available in the NCI 60 cell lines. The 150 drug-CCRG pairs included 64 drugs and 94 genes. A total of 47 of 94 genes were detected for their expression in NCI 60 cell lines and 31 of 64 drugs were detected for their activity in NCI 60 cell lines; these 31 drugs and 47 genes comprised 62 drug-CCRG pairs of the original 150 drug-CCRG pairs. We then performed correlation-based analysis on these 62 drug-CCRG pairs. In Figure 2, drug-CCRG pairs whose *PCC* range from −0.3 to 0.3 accounts for 80.6% of all drug-CCRG pairs while drug-CCRG pairs whose *PCC* range from −0.5 to 0.5 accounts for 91.9% of all drug-CCRG pairs. Thus when we identify the drug-candidate CRGs with high *PCC* (*PCC*0.3% = 0.39, *PCC*0.5% = 0.51, both *PCC* thresholds are set in concordance with previous studies [40,41]), the *PCC*s of the majority of drug-CCRG pairs fall below the cut off threshold.

**Figure 3:**
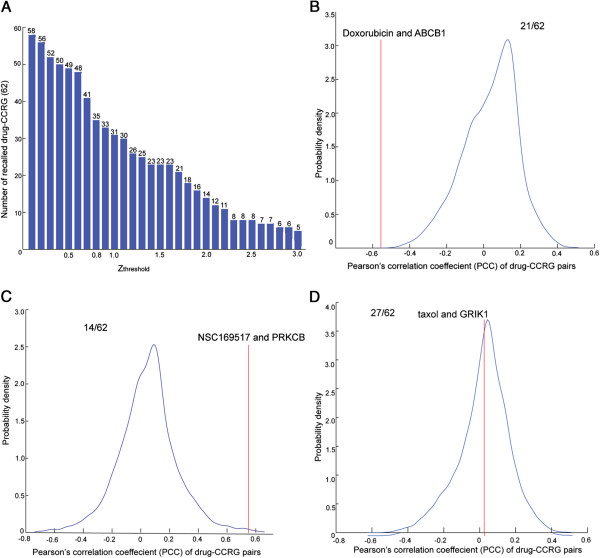
**Correlation-based analysis of the drug-CCRG pairs.** (**A**) For each of the 62 drug-CCRG pairs, we compared the *PCC* of drug-CCRG with that of random drug-gene pairs. The numbers of drug-CCRG pairs, which were identified under the corresponding *z*_*threshold*_, were listed over the blue bar. We set *z*_*threshold*_ to 0.8 in concordance with previous reports (Proc Natl Acad Sci U S A 2001, 98:10787–10792). Under this threshold, we conducted further analysis (Figure 3B, Figure 3C, and Figure 3D). (**B**, **C**, **D**) Three types of *PCC* distribution compared to random *PCC*. The *x*-axis shows the *PCC* of drug-gene pair, the y-axis shows the probability density value of *PCC*. The red line represents the *PCC* of a drug-CCRG pair, while the blue curves shows the distribution of *PCC* of random drug-gene pairs. (**B**) *PCC* of drug-CCRG is significantly smaller than *PCC* of random drug-gene pairs. 21/62 indicates that 21 of 62 drug-CCRG pairs exhibit *PCC*s significantly smaller than random *PCC*s. We offered an example between doxorubicin and ABCB1. It was reported that ABCB1 overexpression predicts doxorubicin resistance. (**C**) *PCC* of drug-CCRG is significantly larger than that of random drug-gene pairs. 14/62 indicates that 14 of 62 drug-CCRG pairs exhibit *PCC*s significantly largerr than random *PCC*s. It was reported PRKCB can predict chemosensitivity of NSC169517. (**D**) *PCC* of drug-CCRG is similar with that of random drug-gene pairs. 27/62 indicates that 27 of 62 drug-CCRG pairs do not exhibit *PCC*s significantly different from random *PCC*s. It was reported that GRIK1 was able to predict chemosensitivity of paclitaxel (taxol).

Although the *PCCs* of drug-CCRG pairs are not high, they may be significantly larger than random genes. Thus, for each of the 62 drug-CCRG pairs we determined whether the *PCC* was significantly larger or smaller than random *PCC*. We found that *PCC* of certain drug-CCRG pairs was significantly smaller than random pairs, whereas *PCC* of certain drug-CCRG pairs was significantly larger. There were also some pairs with *PCC* similar to random drug-gene pairs. The comparisons of drug-CCRG *PCC* with random *PCC* are shown in Additional file 2 for each of the 62 drug-CCRG pairs. We calculated how many pairs of drug-CCRG exhibited significant larger or smaller *PCC* than random *PCC*. The statistical method we used was *z*_*i*_ = |*x*_*i*_ − *μ*|/*δ*, where *x*_*i*_ is the *PCC* of drug-CCRG pair *i*, and *μ* and *δ* are the mean and standard deviation of all the *PCC* for the drug in this drug-CCRG pair. Figure 3A shows the number of identified drug-CCRG pairs under different thresholds. If *z*_*i*_ *≥ z*_*threshold*_, the *PCC* of drug-CCRG pair *i* is significantly different from random *PCC*. The numbers of drug-CCRG pairs, which were identified under the corresponding *z*_*threshold*_, were listed over the blue bar. As the stricter *z*_*threshold*_ was, fewer drug-CCRG pairs were identified. For example, when using 1 as the *z*_*threshold*_, only 32 of 62 drug-CCRGs were identified, whereas when using 2 as the *z*_*threshold*_, only 15 of 62 were identified, and when using 3 as the *z*_*threshold*_ only 6 of 62 were identified. As shown in Figure 3A, we found it was not sufficient to identify drug-CCRG pairs using *PCC* based on random analysis. We set the threshold to 0.8 in concordance with the previous reports [17]. Among the 62 drug-CCRG pairs, 21 pairs exhibit smaller *PCC* than random drug-gene pairs (Figure 3B), 14 pairs exhibit larger *PCC* than random drug-gene pairs (Figure 3C) and 27 pairs exhibit random *PCC* (Figure 3D).

Figure 2 and Figure 3 show that the majority of drug-CCRGs exhibit a low correlation between gene expression and drug activity. Moreover, 27/62 (44%) of drug-CCRG correlations tend to be random by comparing *z*_*i*_ with *z*_*threshold*_. Thus we investigated to integrate additional functional information to predict drug-CRGs.

### GO enrichment analysis of CCRGs

CCRGs are significantly enriched in 204 terms (*p* < 0.01) according to Fisher’s exact test. For a complete list of enriched GO terms, see Additional file 3. The majority of enriched GO terms are related to chemosensitivity. For example, the GO terms “basolateral plasma membrane” are related to chemosensitivity linked by ABCB5 [42]. First-pass elimination of CRC 220 is due to an active carrier-mediated transport process in the “basolateral plasma membrane” [43]. Lesions in oncogenes and tumour suppressor genes involved in “the regulation of programmed cell death” appear to be important in the evolution of drug resistance [44]. Proteins involved in “regulation of apoptosis” are associated with cisplatin chemosensitivity in germ cell tumors [45]. Genes involved in “regulation of cell cycle”, such as p53 protein family, contribute to chemotherapeutic drug response in gastrointestinal tumors [46]. “Xenobiotic metabolism” involves modifying the chemical structure of xenobiotics, such as drugs and poisons. Reactions in these pathways contribute to chemosensitivity in cancer. Furthermore, CCRG enriched GO terms exhibit significantly greater similarity compared to randomly selected genes. This indicates that CCRG enriched GO terms are more similar to each other when compared with GO terms where random genes enriched (Additional file 4).

### The characteristics of CCRGs in PPIN

Degree of a gene in PPIN is characterized by the number of its adjacent genes. It depicts the importance of the gene in maintaining the connectivity of PPIN, and a gene with high degree is called a hub. The average degree of CCRGs was significantly smaller compared to random genes in corresponding networks (Table 2). This indicates that CCRGs tended to connect with many other genes compared to random genes, suggesting that CCRGs play key roles in maintaining the connectivity of PPIN.

**Table 2:** Degree of CCRG compared with random genes

	**mean of CCRG**	**mean of random genes**	fold*	***p *value**^**#**^
BIND	9.24444	3.65349	2.53031	0.002
IntAct	22.01515	7.31961	3.0077	<0.001
MINT	9.77358	5.26234	1.85727	0.021
HPRD	26.10526	7.71614	3.3832	<0.001
BioGRID	24.86486	7.03614	3.53388	<0.001
OPHID	43.71429	12.2352	3.57283	<0.001

*fold is the result of mean degree of CCRG divided by that of random genes.

^#^1000 random gene sets was selected from network, each randomization kept the number of selected genes same as that of real genes. Then for CCRG, *p* value was calculated over 1000 randomization.

Betweenness centrality is a global centrality index that quantifies the extent that a gene controls the information flow between all pairs of genes in the network. Table 3 shows that in all of the networks the mean betweenness centrality of CCRGs is significantly larger compared to random genes in the network. Genes with high betweenness centrality controls most of the information flow in the network, and represent the critical points of the network. These genes are called the “bottlenecks” of the network. This indicates that CCRGs play key roles in controlling information flow of PPIN.

**Table 3:** Betweenness centrality of CCRG compared with random genes

	**mean of CCRG**	**mean of random gene**	fold^**^	***p *value**^**#**^
BIND	98654.40138	23213.11683	4.24994	<0.001
IntAct	149690.9051	36049.94851	4.15232	0.008
MINT	93562.07001	27842.49937	3.36058	0.015
HPRD	214315.4538	40236.19003	5.32644	<0.001
BioGRID	281377.0414	51171.666	5.49869	0.01
OPHID	252315.597	39821.59949	6.33615	<0.001

^**^fold is the result of mean betweenness centrality of CCRG divided by that of random genes.

^#^1000 random gene sets was selection from network, each randomization kept the number of selected genes same as that of real genes. Then for CCRG, *p* value was calculated over 1000 randomization.

### Performance of the proposed method to identify drug-CRGs

Here, we used hypergeometric tests to evaluate the extent to which predicted drug-CRGs appeared in the drug-CCRGs. The significance of the over-representation was calculated by the hypergeometric test:

$$P=\sum_{x\geq n}\frac{C_{N}^{x}C_{M-N}^{m-x}}{C_{M}^{m}}$$

where *M* was the total number of all drug-candidate CRGs; *N* was the number of predicted drug-CRGs using our method; *m* was the number of drug-CCRGs; *n* was the number of drug-CCRGs correctly predicted by our method. In order to ensure the comparability of our method and the method based on gene expression, we keep number of predicted drug-CRG pairs obtained by both methods equal with each other. Using different thresholds for betweenness centrality, degree and *PCC*, we obtained different numbers of drug-gene pairs. In order to identify the greatest number of drug-CCRG pairs, we set the *PCC* threshold to the fifth percentile (5%) of *PCC* for all drug-CCRG pairs. We compared the performance of both methods under 20 sets of thresholds for betweenness centrality and degree; the results are shown in Table 4. The proposed method identified a greater number of drug-CCRGs under all of the thresholds. Moreover, drug-CCRGs were much more significantly enriched in the drug-CRGs predicted by our method.

**Table 4:** Performance of our method to predict drug-CRGs under different thresholds

Threshold of degree^*^	**Threshold of betweenness centrality**	**The proposed method**		**Method based on gene expression**	
		**Number of identified CCRGs**	**enrichement significance**	**Number of identified CCRGs**^**#**^	**enrichement significance**
0.01	0.01	6	1.20E-08	5	5.34E-06
0.02	0.02	8	0.00	6	1.05E-05
0.03	0.03	10	0.00	7	5.76E-06
0.04	0.04	11	0.00	7	2.87E-05
0.05	0.05	11	0.00	7	1.20E-04
0.06	0.06	11	0.00	8	4.56E-05
0.07	0.07	13	0.00	8	1.17E-04
0.08	0.08	13	0.00	8	2.61E-04
0.09	0.09	13	7.46E-08	8	5.47E-04
0.1	0.1	13	2.01E-08	8	8.87E-04
0.11	0.11	13	1.08E-08	9	3.05E-04
0.12	0.12	14	6.10E-08	9	5.71E-04
0.13	0.13	14	0.00	11	5.07E-05
0.14	0.14	14	3.60E-07	11	7.91E-05
0.15	0.15	14	7.63E-07	11	1.30E-04
0.16	0.16	14	1.79E-06	11	2.40E-04
0.17	0.17	14	2.80E-06	11	3.26E-04
0.18	0.18	14	5.26E-06	11	5.14E-04
0.19	0.19	14	7.77E-06	11	6.89E-04
0.2	0.2	15	3.25E-06	11	1.13E-03

^*^Degree threshold is set to one percentile (0.01) of degree for all the genes in HPRD. And 0.02 represents that we set the degree threshold as two percentile of degree for all the genes in HPRD. Betweenness centrality threshold is set in the same way as degree threshold.

^#^In order to ensure the comparability of our method and the method based on correlation of gene expression and drug activity, we kept the number of drug-CRGs obtained by both methods equal with each other.

We next evaluated the performance of the proposed method by ROC to determine whether CCRGs were distinguished from other genes. For the proposed method, we ranked all of the genes in predicted drug-CRGs using the *Q* statistic (See details in Methods) in order to integrate various separate data sources. We integrated ranks of degree and betweenness centrality to determine whether CCRGs ranked at the top of the list. According to *Q* statistics and whether genes were CCRGs, we plotted the ROC curves. For traditional correlation method, we ranked all drug-CRG pairs using absolute *PCC* of gene expression and drug activity. According to *PCC* and whether genes were CCRGs, we also plotted the ROC curves.

**Figure 4:**
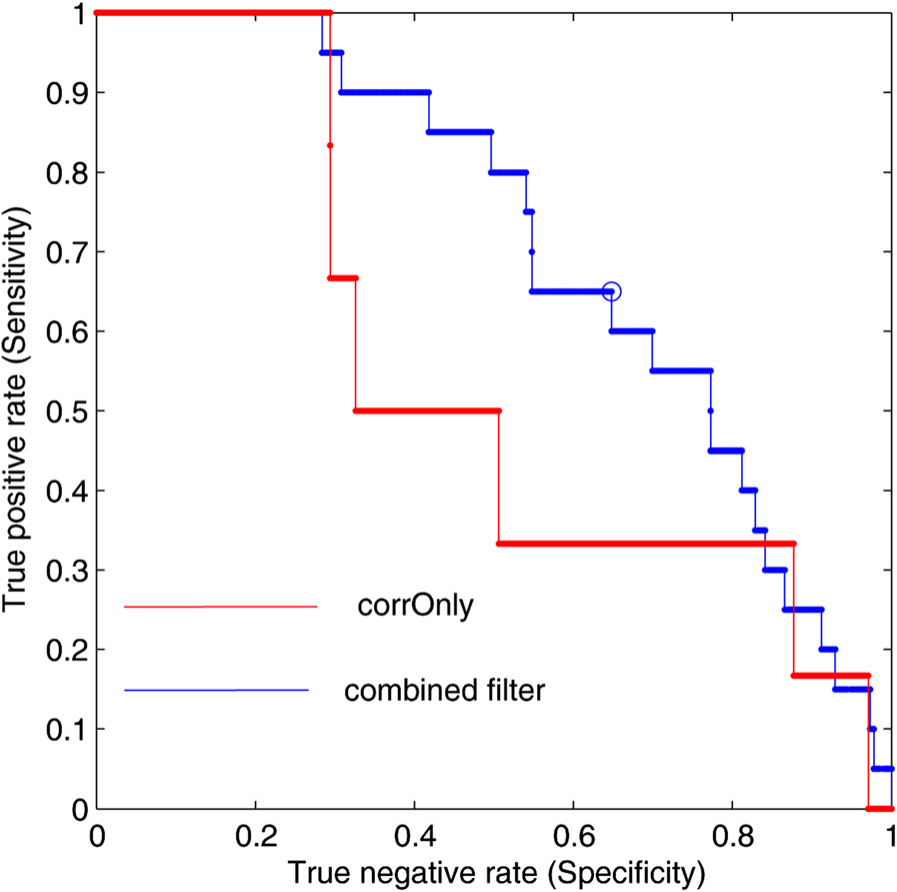
**The ROC curve was created by plotting the sensitivity against specificity.** Sensitivity is the fraction of number of true positive assessment versus that of all positive assessment (Sensitivity = TP/ (TP + FN)). Specificity is the fraction of number of true negative assessment versus that of all negative assessment (Specificity = TN/ (TN + FP)). The red line “corrOnly” represents the traditional method to identify CRGs only based on the correlation between gene expression and drug activity. The blue line “combined filter” represents the proposed method to identify CRGs by integrating information from CCRG enriched GO terms and network features of PPIN. The ROC curve was used to evaluate the performance of both methods. For the proposed method, we rank all the genes in HPRD protein interaction network by *Q* statistics (see details in the Methods section of the manuscript). According to *Q* statistics and whether the genes are CCRGs, we plotted the ROC curves for our method. While for the traditional correlation method, we ranked all drug-CRG pairs using absolute *PCC*. According to *PCC* and whether genes were CCRGs, we also plotted the ROC curves.

Our findings indicated that our approach was almost exclusively superior to the traditional method based on gene expression. The mean area under ROC curve (AUC) for our method is 65.2%, whereas that for the traditional method AUC is 55.2%. In Figure 4, AUC was 0.5446 for the correlation coefficient method based on gene expression whereas the AUC achieved up to 0.7087 for our method. Detailed performance comparison under all the 20 thresholds, see Additional file 5.

### Identification of CRGs by integrating CCRGs’ properties in GO and PPIN

Based on gene expression, GO categories, and network characteristics, we identified CRGs for drugs. Combined filtering method is superior compared with the method using only Pearson’s correlation coefficients based on gene expression. We used this combined filtering method to identify CRGs for all of the drugs, whose activities were screened in NCI 60 cell lines. Consequently, we obtained 53 genes that were not only associated with chemosensitivity related GO categories but also played key roles in maintaining connectivity and controlling the information flow of PPIN. Among the 53 CRGs, 32 were previously reported as chemosensitivity related genes. The full gene list is in Additional file 6.

Our findings are supported by previous studies. Genes with high correlation coefficients are identified as CRGs. For example, *EGFR* is negatively correlated with activity of Tamoxifen, and the Pearson’s correlation coefficient (*PCC*) is – 0.39. This suggests that expression of *EGFR* can predict the resistance to Tamoxifen, which is consistent with a previous study in which *EGFR* product resulted in decreased susceptibility to Tamoxifen [47]. At the same time, *BRCA1* is positively correlated with activity of Tamoxifen (*PCC* = 0.25); this indicates that *BRCA1* expression can predict sensitivity of Tamoxifen, which is in concordance with a previous study in which the overexpression of *BRCA1* results in increased susceptibility to Tamoxifen[48]. We also identified candidate CRGs with low *PCC*. For example, although *AKT1* is weakly correlated with sensitivity of Doxorubicin (*PCC* = 0.13), it has been reported to result in increased susceptibility to Doxorubicin [49]. *EGFR* product affects the susceptibility to Fluorouracil (*PCC=*– 0.2) [50], *RB1* affects the susceptibility to Fluorouracil (*PCC*=– 0.09) [51], *RELA* product affects the susceptibility to Doxorubicin (*PCC*=– 0.05) [52], STAT3 affects the susceptibility to Fluorouracil (*PCC*=– 0.18) [53], and *TP53* product affects the susceptibility to Fluorouracil (*PCC* = 0.04) [54]. These results indicate that these genes exhibit the potential to predict chemosensitivity of drugs before initiating therapy, which could potentially aid clinical decisions and allow for more individualized treatment strategies for patients.

## Discussion

The high-resolution profiling at the mRNA level and high-throughput drug sensitivity data of NCI 60 allow for comprehensively mapping of mRNA profiles for molecular pharmacologic and drug discovery [55]. There are previously reported high-throughput studies on CRG identification for drugs; however, most of these studies are based on gene expression. Some studies reported genes with expression levels highly correlated with drug activity as CRGs, chemosensitivity genes with low *PCC* were excluded. Aside from correlation analysis, some researchers have developed other computational methods based on gene expression. However, individual genes were studied in isolation rather than in the context of their functional interactions. In fact, genes are not functionally independent; they work in synergy to perform biological function.

In our proposed method, we utilized high-throughput gene expression profiles to predict CRGs by integrating drug-gene correlations, gene function annotation, and network information. We systematically characterized CCRGs in the context of functional genomic data; we then prioritized CRGs based on these CCRG characteristics. Firstly, we conducted an extensive literature survey and manually curated a compendium of CCRGs. According to GO analysis on three ontologies, most of the CCRG enriched GO terms were related to chemosensitivity. Moreover, these GO terms were more similar to each other compared to randomly selected genes. CCRGs also play key roles in protein-protein interaction network (PPIN). They control the information flow of PPIN and maintain connectivity of PPIN. The initial drug-candidate CRG network was pruned according to these characteristics; consequently we obtained a database of predicted drug-CRGs for all drugs whose activity profiles were screened in NCI 60 cell lines. The results demonstrated that our method can not only identify CRGs whose expression is strongly correlated with drug activity, but also can identify CRGs whose expression is weakly correlated with drug activity. These results are powerfully supported by previous studies. From the predicted drug-CRGs, the researchers can easily access genes and drugs of interest, thus facilitating further studies. Functional genomic information, such as GO categories and protein interaction networks, aid the identification of CRGs unable to be identified by methods based only on similarity between gene expressions and drug activity.

The present analysis has the following limitations: (a) the drug-CCRGs we curated are limited to NCI 60 data. (b) the data presented here give an incomplete biological picture of the relationship between drug and CRG. Further validation of drug-CRG relationships is necessary prior to clinical application. (c) the conclusions were extrapolated from in vitro to in vivo. Transformed cell lines might further evolve in vitro and might not reflect the tumor from which they were originally isolated. (d) finally, the relationships established between drug activities and gene expression levels are correlative, not causal.

## Conclusions

In summary, we provide an integrated method of identifying CRGs that combines gene expression, drug activity data and functional information for genes such as GO categories and PPIN. We documented 150 pairs of drug-CCRG from 492 published papers. CCRG enriched GO terms were generally related to chemosensitivity. These GO terms exhibited higher similarity compared to GO terms enriched by randomly selected genes. Moreover, CCRGs play key roles in maintaining connectivity and controlling information flow of PPIN. Thus, we pruned the initial drug-candidate CRG network based on CCRG GO categories and network characteristics. As a result, we obtained a database of predicted drug-CRGs. It includes 53 CRGs, 32 of which have been previously reported to be chemosensitivity related genes.

The CRGs identified will potentially allow for greater treatment efficacy and fewer unnecessary side effects. For patients predicted not to respond to certain agent, alternative agents or combined agents could be considered. Candidate second-line anticancer drugs for combination therapy may be selected based on the database of predicted drug-CRGs. Moreover, the CRGs may serve as candidate drug targets for the development of new drugs. With additional validated drug-CCRG pairs, our proposed method could potentially provide valuable resources for pharmacogenomics research and contribute to the framework for individualized medicine.

## Competing interests

The authors declare that they have no competing interests.

## Authors’ contributions

XC, QHW and XL conceived this study. WJ made critical contributions to manuscript revisions. XC performed the literature survey. WJ and PW conducted pre-experiment. XC, TH and YL analyzed the data and performed statistical analysis. XC and WJ drafted the manuscript. XWC and YLL contributed to manuscript editing. All authors read and approved the final manuscript.

## Pre-publication history

The pre-publication history for this paper can be accessed here:

http://www.biomedcentral.com/1755-8794/5/43/prepub

## Supplementary Material

Additional file 1 **Table S4.** Detailed description of drug-CCRG pairs. Click here for file

Additional file 2 **Figure S1.** The comparison of drug-CCRG *PCC* with random *PCC* for each of the 62 drug-CCRG pairs. Each subfigure of this figure shows the location of Pearson's correlation coefficient (*PCC*) of a drug-CCRG pair in all the drug-gene pairs. The red line represents the *PCC* of a drug-CCRG pair, while the blue curves shows the distribution of *PCC* of all the drug-gene pairs. The x-axis shows the *PCC* of drug-gene pair. The y-axis shows the probability density value of *PCC*. Click here for file

Additional file 3 **Table S1.** A full list of CCRG enriched GO terms. Click here for file

Additional file 4 **Table S2.** CCRG enriched GO terms have higher similarity than that of random genes. Click here for file

Additional file 5 **Figure S2.** Detailed performance comparison under all the 20 thresholds. Figure A to Figure T shows the comparison result of two methods to identify CCRGs under 20 sets of thresholds. A is the result under the following threshold: degree_threshold: percentile 1 (0.01), betweenness centrality_threshold: percentile 1(0.01). B is the result under the threshold: degree_threshold: percentile 2 (0.02), betweenness centrality_threshold: percentile 2 (0.02). And the corollary, Figure T is the result under the threshold: degree_threshold: percentile 20 (0.20), betweenness centrality_threshold: percentile 20 (0.20). The text over each figure is the area under curve (AUC). Take Figure A for example, 0.7087 vs 0.5446 represents that AUC of our method is 0.7087, and 0.5446 is AUC of traditional method based on gene expression. The AUC is colored according to curve color. Click here for file

Additional file 6 **Table S3.** The full list of predicted chemosensitivity related genes. Click here for file
