# Supplementary figures and images for: Systematically characterizing and prioritizing chemosensitivity related gene based on Gene Ontology and protein interaction network
Source: BMC Med Genomics. 2012 Oct 2;5:43. doi: 10.1186/1755-8794-5-43 (PMC3532125; doi:10.1186/1755-8794-5-43)

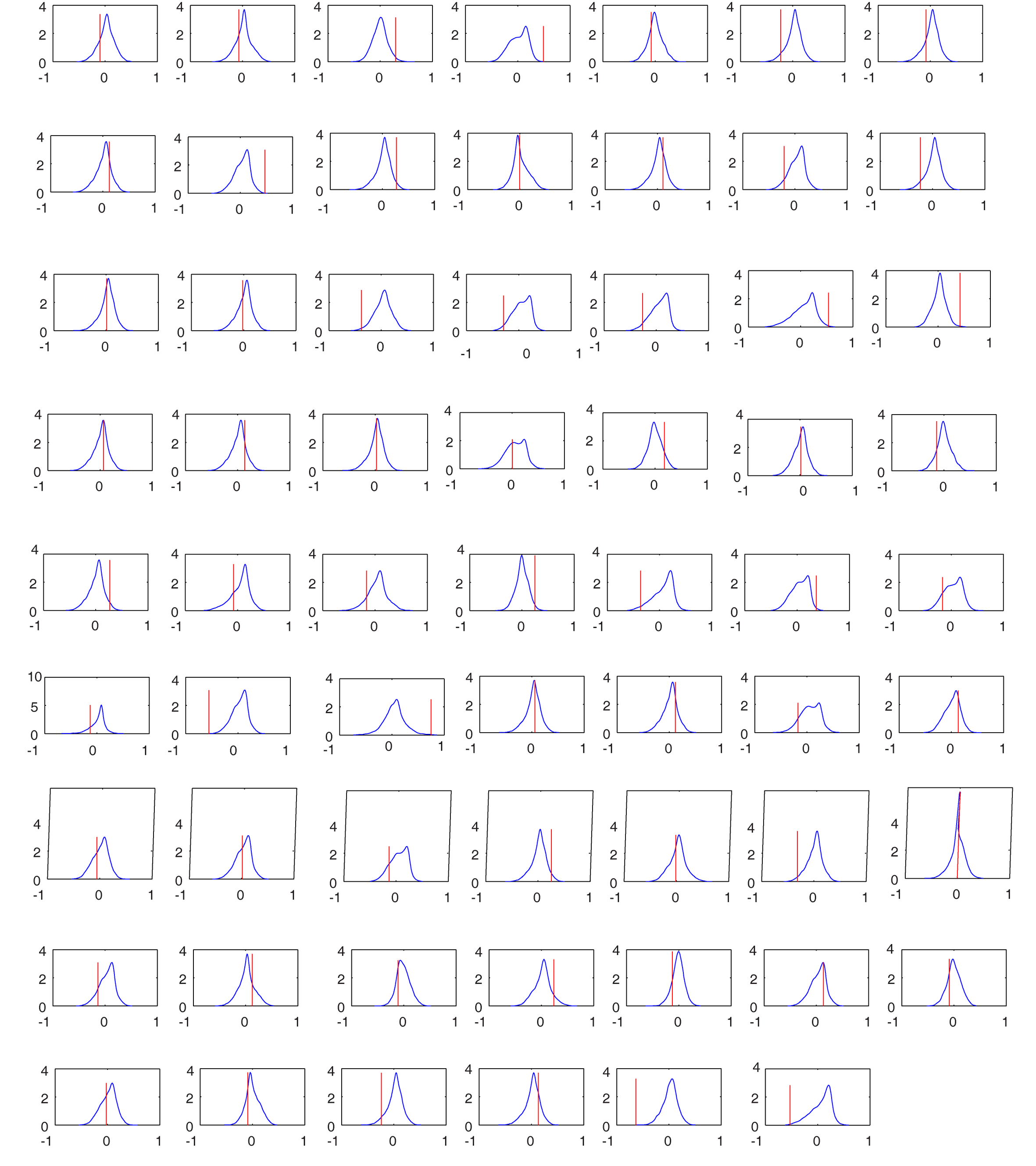

Supplement: Additional file 2 — Figure S1. The comparison of drug-CCRG PCC with random PCC for each of the 62 drug-CCRG pairs. Each subfigure of this figure shows the location of Pearson's correlation coefficient (PCC) of a drug-CCRG pair in all the drug-gene pairs. The red line represents the PCC of a drug-CCRG pair, while the blue curves shows the distribution of PCC of all the drug-gene pairs. The x-axis shows the PCC of drug-gene pair. The y-axis shows the probability density value of PCC. [file 1755-8794-5-43-S2.tiff]

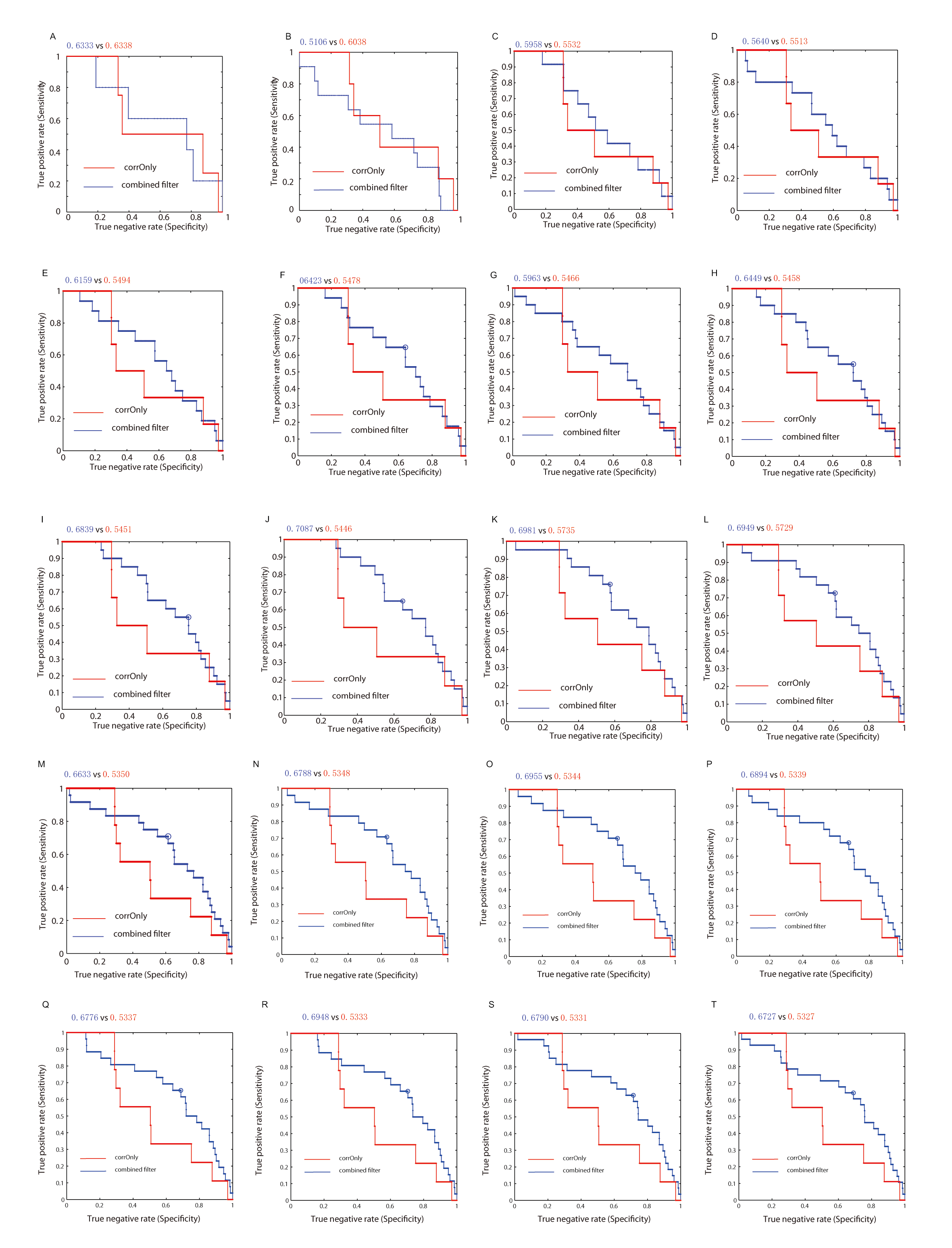

Supplement: Additional file 5 — Figure S2. Detailed performance comparison under all the 20 thresholds. Figure A to Figure T shows the comparison result of two methods to identify CCRGs under 20 sets of thresholds. A is the result under the following threshold: degree_threshold: percentile 1 (0.01), betweenness centrality_threshold: percentile 1(0.01). B is the result under the threshold: degree_threshold: percentile 2 (0.02), betweenness centrality_threshold: percentile 2 (0.02). And the corollary, Figure T is the result under the threshold: degree_threshold: percentile 20 (0.20), betweenness centrality_threshold: percentile 20 (0.20). The text over each figure is the area under curve (AUC). Take Figure A for example, 0.7087 vs 0.5446 represents that AUC of our method is 0.7087, and 0.5446 is AUC of traditional method based on gene expression. The AUC is colored according to curve color. [file 1755-8794-5-43-S5.tiff]
