# Supplementary material for: Systematically characterizing and prioritizing chemosensitivity related gene based on Gene Ontology and protein interaction network
Source: BMC Med Genomics. 2012 Oct 2;5:43. doi: 10.1186/1755-8794-5-43 (PMC3532125; doi:10.1186/1755-8794-5-43)
Supplement: Additional file 4 — Table S2. CCRG enriched GO terms have higher similarity than that of random genes. [file 1755-8794-5-43-S4.doc]

Table S2 CCRGs enriched in GO terms with higher similarity

|  | enriched term  set similarity* | random term set similarity | fold of similarity  (CCRG/random) | *p* value |
| --- | --- | --- | --- | --- |
| BP-1 | 4.763360569 | 4.59126295 | 1.037483721 | 0.165 |
| BP-2 | 7.841207898 | 6.20553737 | 1.263582416 | <0.005 |
| BP-3 | 9.468104134 | 6.4027355 | 1.478759218 | <0.005 |
| BP-4 | 10.1540088 | 6.56144268 | 1.547526862 | <0.005 |
| BP-5 | 11.2065688 | 6.47581789 | 1.730525625 | <0.005 |
| MF-1 | 3.224983443 | 4.06081228 | 0.794172009 | 0.925 |
| MF-2 | 7.130407841 | 4.8006631 | 1.485296447 | <0.005 |
| MF-3 | 9.482255399 | 5.6210522 | 1.686918224 | <0.005 |
| MF-4 | 11.288934 | 5.31083664 | 2.125641357 | <0.005 |
| MF-5 | 9.816308782 | 5.0877045 | 1.929418027 | <0.005 |
| CC-1 | 3.456894625 | 2.76765466 | 1.249033947 | <0.005 |
| CC-2 | 4.333211773 | 3.90003277 | 1.111070605 | 0.055 |
| CC-3 | 5.666589478 | 4.37185854 | 1.29615115 | <0.005 |
| CC-4 | 5.781969391 | 4.47090976 | 1.293242247 | <0.005 |
| CC-5 | 5.666608925 | 4.43261552 | 1.278389452 | <0.005 |

Fisher exact test was used to perform GO enrichment. If enriched p value is smaller than 0.01, the genes are significantly enriched in the GO term. The first column depicts function aspects of the Gene Ontology and the annotation depth. Three aspects of GO are biological process (BP), molecular function (MF) and cellular component (CC), respectively. The second column depicts average similarity of enriched term sets, which is marked *. It’s described detailed in the section of “**Semantic similarity of GO terms**”. The third column depicts average similarity of enriched term sets when randomly selected genes from whole human genome with the same number of CCRG. The forth column depicts the fold change of similarity between enriched GO term sets of CCRG and random genes. It is the result of column 2 divided by column 3. The last column depicts the location of average similarity of CCRG enriched term sets in the random condition.

From the result, it’s indicated that GO terms in which CCRG enriched in are more similar to each other when compared with GO terms where random genes enriched in. p value is calculated by 200 randomizations.

**Semantic similarity of GO terms**

Fisher Exact test is used to measure the CCRG enriched GO terms. If the p≤0.01, the term is significantly enriched by CCRGs.

Yang et al. investigated the functional consistence (or stability) of threshold-dependent methods based on semantic similarity of GO categories[1]. Under various differentially expressed genes (DEG) thresholds, the results show that the DEGs are functionally consistent. The semantic similarity measure we used was Jiang’s term similarity measures[2] and best-match average (BMA).

Given two terms *c*_1_ and *c*_2_, and their most informative common ancestor *c_A_*, Jiang and Conrath's similarity measure is given by the following equation:

$$sim\left(c_{1},c_{2}\right)=1+IC\left(c_{A}\right)-\frac{IC\left(c_{1}\right)+IC\left(c_{2}\right)}{2}$$

where$IC\left(c\right)=-\log p\left(c\right)$, *p*(*c*) is the probability of using term *c* in the universal term set. To calculate this frequency, we first count the number of distinct proteins annotated to term *c* or one of its descendent terms, and then divide the number by the total number of proteins annotated within the corresponding GO domains.

Given two non-redundant sets of GO term annotation GO(*A*) and GO(*B*), respectively. The best-match average approach is given by the average similarity between each term in GO(*A*) and its most similar term in GO(*B*), averaged with its reciprocal to obtain a symmetric score:

$$sim_{BMA}\left(A,B\right)=\frac{AVG_{t_{1}}\left(MAX_{t_{2}}sim\left(t_{1},t_{2}\right)\right)+AVG_{t_{2}}\left(MAX_{t_{1}}sim\left(t_{1},t_{2}\right)\right)}{2}, t_{1}\in GO\left(A\right), t_{2}\in GO\left(B\right)$$

*t*_1_ and *t*_2_ represent any terms in term sets GO(*A*) and GO(*B*), respectively.

1. Yang D, Li Y, Xiao H, Liu Q, Zhang M, Zhu J, Ma W, Yao C, Wang J, Wang D, et al: **Gaining confidence in biological interpretation of the microarray data: the functional consistence of the significant GO categories.** *Bioinformatics* 2008, **24:**265-271.

2. Jiang JJ, Conrath DW: **Semantic Similarity Based on Corpus Statistics and Lexical Taxonomy.** *Proc of the 10th International Conference on Research on Computational Linguistics* 1997.
